# Screening for Success: Turning the Tables on Colon Cancer Before It Strikes

**DOI:** 10.7759/cureus.106953

**Published:** 2026-04-13

**Authors:** Cleon Rogers, Meghan Pattison, Byron Berry, Willa Green, Butler Wilbanks, Mason Hemstreet, Warren S Hancock, Natalie Pang, Zoe Tenner, Estefania R Perez, Kristina Snoddy, Chandler Pugh, Shirlyncia Moore, Tamia L Soto, Bradley W Cantley

**Affiliations:** 1 Internal Medicine, Christ Health Center, Birmingham, USA; 2 Family Medicine, Christ Health Center, Birmingham, USA; 3 Medical School, Edward Via College of Osteopathic Medicine, Auburn, USA; 4 Medicine, Edward Via College of Osteopathic Medicine, Auburn, USA; 5 Department of Physician Assistant Studies, Samford University, Homewood, USA

**Keywords:** colon cancer prevention, colon cancer surveillance, colorectal cancer, colorectal cancer screening and detection, federally qualified health center, medically underserved populations, patient outreach, quality measures, screening guidelines, stool dna testing

## Abstract

Background

Colorectal cancer is the second leading cause of cancer-related death in the United States. Screening has been shown to reduce incidence and mortality, yet adherence remains suboptimal, particularly among underserved populations.

Objective

This study aimed to evaluate adherence to colorectal cancer screening guidelines among patients aged 45-75 years at Christ Health Center (CHC), a Federally Qualified Health Center in Birmingham, Alabama.

Methods

Patients aged 45-75 years without documented colorectal cancer screening in the preceding year were identified using the Azara Data Reporting and Visualization System. A total of 2,061 patients were initially identified; 191 were excluded due to death, relocation, unstable medical conditions, or receipt of psychiatric-only care, resulting in 1,870 patients eligible for review. Screening status was subsequently verified through the electronic medical record (EMR), and eligible patients were contacted up to three times to encourage completion of screening. Available screening modalities include colonoscopy, fecal immunochemical testing (FIT), and stool DNA testing (Cologuard). Outcomes were recorded in a secure, HIPAA-compliant database. Patients found to be up to date with colorectal cancer screening despite inaccurate EMR documentation were excluded, yielding a final analytic cohort of 1,680 patients.

Results

The mean patient age was 67.3 years, and 1,179 (62.2%) were female. A total of 1,046 (55.9%) could not be reached despite multiple attempts. Among 585 (31.3%) contacted patients, 319 (54.5%) agreed to screening while 266 (45.5%) declined. In addition, 243 (13%) had prior screening that was not mapped appropriately within the EMR. The group most frequently lacking up-to-date screening was females aged 65-75 years, while the lowest percentage occurred among newly eligible patients aged 45-55 years.

Conclusion

Direct patient outreach significantly improved screening uptake among eligible patients, demonstrating the effectiveness of proactive communication in a community health setting. However, barriers such as incomplete EMR documentation, patient engagement challenges, and insurance limitations persist. Enhanced outreach efforts, improved record integration, and targeted education strategies are needed to strengthen colorectal cancer screening adherence in underserved populations.

## Introduction

Christ Health Center (CHC) is a Federally Qualified Health Center (FQHC) located in Birmingham, Alabama, which serves as a critical resource for underserved populations. FQHCs provide primary care services in communities that are classified as medically underserved [1]. In 2023, 4,026 (23%) of the 17,504 patients at CHC were at or below 100% of the poverty guideline, 5,251 (30%) were uninsured, and 438 (2.5%) were homeless [2-3]. According to the Health Resources and Services Administration (HRSA), 13,223 (75.5%) of CHC patients are of known ethnic minorities. Patients facing multiple health disparities, such as those at CHC, are at a disadvantage within the healthcare system for many reasons. Cancer screening is an area of limitation for these patients, and in using a colorectal cancer quality measure, we hope to evaluate the limitations and solutions for such patients. 

Colorectal cancer is the third most commonly diagnosed cancer and the second leading cause of cancer-related mortality [4]. Current guidelines recommend colorectal cancer screening for individuals aged 45 to 75 years using methods such as colonoscopy, the fecal immunochemical test (FIT), or stool-based DNA tests like Cologuard (Exact Sciences Corporation) [5-7]. Regular colon cancer screening significantly reduces mortality rates by identifying cancer at treatable stages [8].

In addition to its overall prevalence, colorectal cancer has shown a concerning rise among younger adults. Several large epidemiologic studies have demonstrated increasing incidence in individuals under the age of 50, a trend that has led to heightened awareness and revisions of national screening recommendations [9-10]. This increase in younger cases was a driving factor in the U.S. Preventive Services Task Force lowering the recommended screening age from 50 to 45 years, underscoring the importance of timely and consistent screening efforts in adults beginning at this earlier age.

Despite the proven benefits of screening, numerous challenges prevent patients from undergoing cancer screening. There has been significant research to identify the barriers to improving the numbers of patients who agree and comply with cancer screening, which has led to the development of quality measures and emphasized the critical role of regular visits to address these issues effectively [11-12]. Quality measures are tools that help us measure healthcare outcomes, allowing us to track how well healthcare services are being delivered and whether they meet goals for good care. Colon cancer screening follow-up is a necessary quality measure, but barriers exist within quality measures, such as continued communication, insurance, and lack of well-visits [13-14]. Research has highlighted the importance and benefit of patient education in screening outcomes, but every vulnerable patient population faces unique challenges [15-19]. For example, a FIT test, the sole screening choice for most self-pay patients, must be done annually, which places a greater burden on these patients and has been proven to have lower adherence to screening due to its yearly requirement [20]. Additionally, time and resources are exceptionally limited. For patients in an underserved population, a doctor visit is often due to multiple pressing health issues, leaving little opportunity for discussion of quality measures during the visit [21].

## Materials and methods

This retrospective quality improvement study was conducted at CHC, an FQHC in Birmingham, Alabama. CHC provides care for a medically underserved population, with 23% of patients at or below the federal poverty guideline, 30% uninsured, and 2.5% experiencing homelessness. The study assessed adherence to colorectal cancer screening guidelines among patients aged 45-75 years.

Patients were identified through the Azara Data Reporting and Visualization System, a population health reporting and analytics platform. Eligible participants were active CHC patients aged 45-75 years with no documented colorectal cancer screening in the prior 12 months. Exclusion criteria included patients who were deceased, those with unstable medical conditions precluding screening, individuals seen only for psychiatric services, and patients no longer residing in Alabama or with inactive medical records.

A total of 2,061 patients met the initial inclusion criteria, of whom 191 were excluded, yielding a final cohort of 1,870 patients. Patients who, over the course of the study, were found to have up-to-date screening that had not been updated in the EMR were also excluded, a total of 243. Based on prior research indicating adherence rates of 40-60% in underserved populations, a minimum sample size of 1,500 patients was estimated to provide greater than 80% power at a significance level of α = 0.05 to detect a 5% absolute difference in adherence rates compared with published benchmarks [11,13]. The final analytic cohort of 1,680 patients exceeded this threshold. 

Patient charts were reviewed by the research team to confirm screening status. Those lacking documentation were contacted up to three times by phone. During outreach, patients were informed about the importance of colorectal cancer screening and offered available options. Colonoscopy and stool DNA testing (Cologuard, Exact Sciences) were offered to insured patients, while uninsured patients were offered fecal immunochemical testing (FIT). Orders were placed in accordance with patient preference and insurance status. 

Data were stored in a password-protected Excel database (Microsoft Corp., USA). Information was transmitted through encrypted institutional email accounts and securely stored in Microsoft OneDrive (Microsoft Corp., USA). Patient confidentiality was maintained throughout the study, and outreach was conducted in compliance with the Health Insurance Portability and Accountability Act.

The primary outcome was the proportion of patients who completed or agreed to colorectal cancer screening after outreach. Secondary outcomes included the number and percentage of patients unreachable and the number and percentage who declined screening. Demographic factors such as age and sex were also analyzed in relation to screening status.

Descriptive statistics were used to summarize baseline demographics and outcomes. Categorical variables were reported as frequencies and percentages, while continuous variables were reported as means and standard deviations. A two-tailed p-value less than 0.05 was considered statistically significant. Sample proportions were used to summarize demographic variables (sex at birth and age group) and outcome variables, including outreach outcome options and gap explanations. Pearson's Chi-square was used to test for associations between demographic variables and outcome variables. Effect sizes, along with their 95% confidence intervals, were calculated. All analyses were conducted using SAS version 9.4 (SAS Institute Inc., USA). 

This study was reviewed and approved as exempt by the Institutional Review Board at Samford University (approval number: EXMT-HP-25-S-17).

## Results

**Figure 1:**
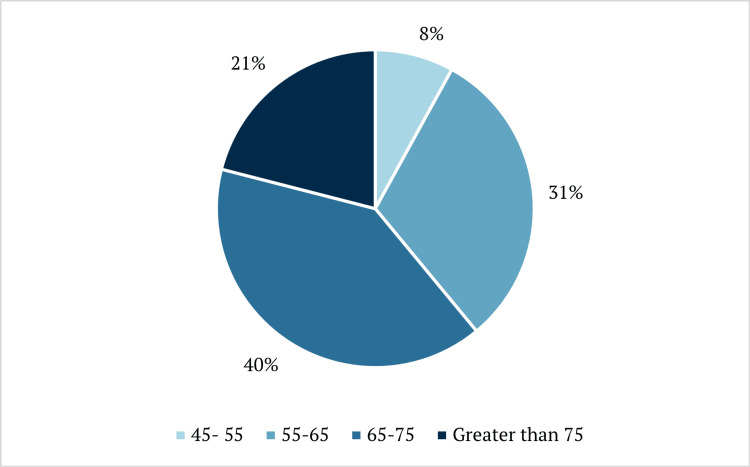
Percentage of patients by age group.

Of the 2,061 patients initially identified, 191 (9.2%) were excluded due to death, unstable medical conditions, relocation, or psychiatric-only care, leaving 1,870 patients for analysis. A further 243 were excluded due to the discovery of up-to-date screening. The mean age of included patients was 67.3 years. The distribution of patients across age groups is shown in Figure 1.

**Figure 2:**
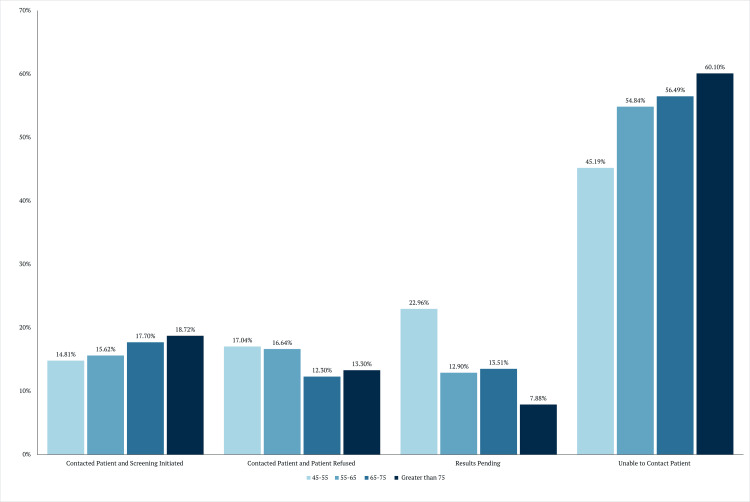
Outcome of contact by age

As shown in Figure 2, the most significant proportion of patients who were unable to be contacted was in the 75+ age group, while the 45-55 age group was the most able to be reached. However, despite the 45-55 age group being more receptive to being contacted, they were less likely to initiate screening than their counterparts. 

**Figure 3:**
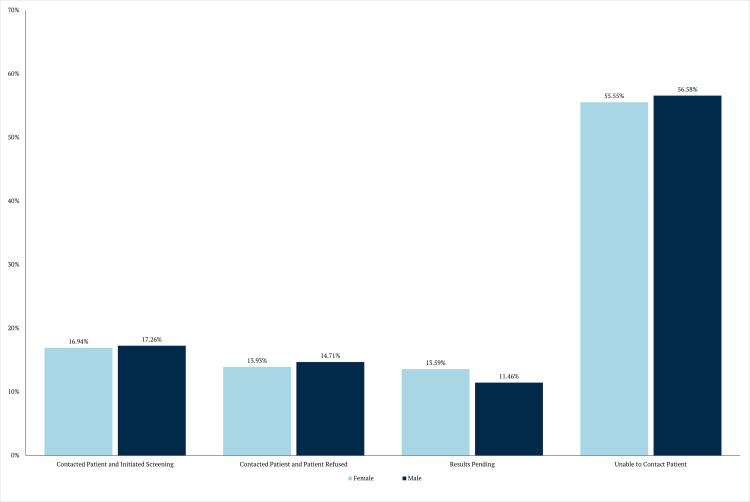
Outcomes of contact by gender

Regarding gender, Figure 3 shows that there was little difference in outcomes between males and females (p = 0.6322). It is essential to note that 1,163 patients (62.2%) were female, and 707 (37.8%) were male. The associations between demographic variables and outreach outcome options are summarized in Table 1.

**Table 1:** Associations for outcome options Legend: a = percent (n), b = p-value from Pearson chi-square test. Significance: *** p < 0.001, ** p < 0.01, * p < 0.05, ns = not significant (p ≥ 0.05).

Variable	Contacted patient and screening initiated	Contacted patient and patient refused	Results pending	Unable to contact patient	p-value	Significance
Values shown as: % (n)						
Sex					0.6027^b^	ns
Female	16.94 (197) a	13.93 (162)	13.59 (158)	55.55 (646)		
Male	17.26 (122)	14.71 (104)	11.46 (81)	56.58 (400)		
Age					0.0004^b^	***
< 55	14.81 (20)	17.04 (23)	22.96 (31)	45.19 (61)		
55–65	15.62 (92)	16.64 (98)	12.90 (76)	54.84 (323)		
65–75	17.70 (131)	12.30 (91)	13.51 (100)	56.49 (418)		
> 75	18.72 (76)	13.30 (54)	7.88 (32)	60.10 (244)		

**Figure 4:**
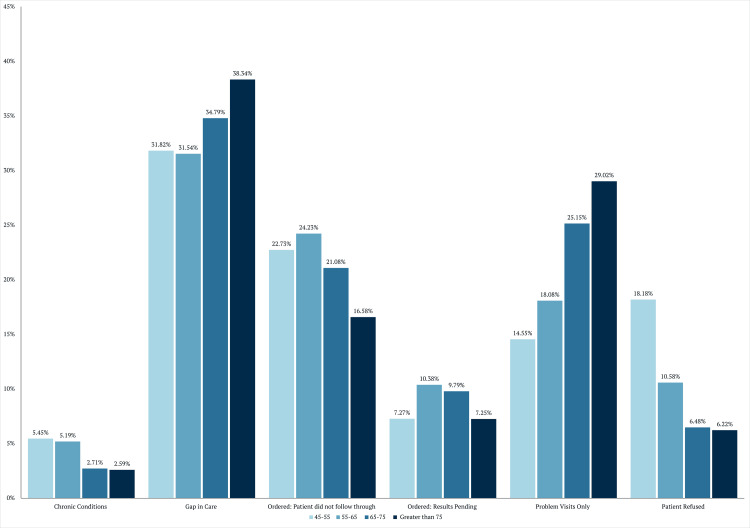
Gap explanations by age

**Figure 5:**
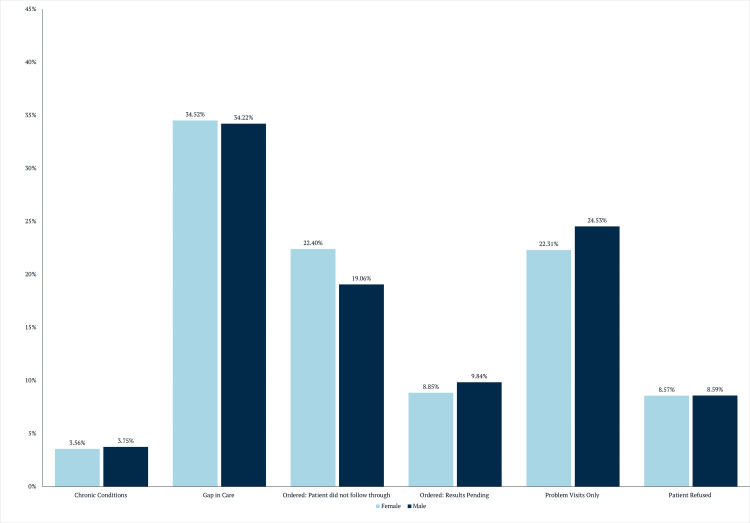
Gap explanations by sex

To further examine the reasons underlying persistent screening gaps, documented care gap explanations were analyzed by demographic group. The distribution of care gap explanations stratified by age group and sex is shown in Figures 4-5.

Outcome categories included serious chronic conditions precluding cancer screening, true gaps in care, screening ordered but not completed, results pending, problem-focused visits only, and patient refusal. A “true gap in care” was defined as a patient who was seen by their primary care physician for a preventive visit during which colon cancer screening was neither discussed nor ordered. In the age-stratified panel, percentages are shown for patients aged 45-55, 55-65, 65-75, and >75 years, demonstrating a progressive increase in the proportion attributed to gaps in care and problem visits only with advancing age, alongside a corresponding decrease in patient refusal and failure to follow through. In the sex-stratified panel, females and males exhibited broadly similar distributions across categories, with modest differences observed in ordered tests not completed and problem-focused visits.

Associations between demographic variables and documented care gap explanations were evaluated using Pearson’s chi-square analysis. The distribution of gap explanations by sex and age group is summarized in Table 2.

**Table 2:** Associations for gap explanations Legend: a = percent (n), b = p-value from Pearson chi-square test. Significance: *** p < 0.001, ** p < 0.01, * p < 0.05, ns = not significant (p ≥ 0.05).

Variable	Chronic conditions	Gap in care	Ordered: patient did not follow through	Ordered: results pending	Problem visits only	Patient refused	p-value	Significance
Values shown as: % (n)								
Sex							0.6322^b^	ns
Female	3.56 (37) a	34.52 (359)	22.40 (233)	8.85 (92)	22.31 (232)	8.57 (87)		
Male	3.75 (24)	34.22 (219)	19.06 (122)	9.84 (63)	24.53 (157)	8.59 (55)		
Age							<0.0001^b^	***
<55	5.45 (6)	31.82 (35)	22.73 (25)	7.27 (8)	14.55 (16)	18.18 (20)		
55–65	5.19 (27)	31.54 (164)	24.23 (126)	10.38 (54)	18.08 (94)	10.58 (55)		
65–75	2.71 (18)	34.79 (231)	21.08 (140)	9.79 (65)	25.15 (167)	6.48 (43)		
>75	2.59 (10)	38.34 (148)	16.58 (64)	7.25 (28)	29.02 (112)	6.22 (24)		

Using a type I error rate of 0.05, Pearson’s chi-square tests did not indicate a statistically significant association between sex at birth and outcome options (χ² = 1.8563, degrees of freedom = 3, p = 0.6027) or between sex at birth and gap explanations (χ² = 3.4418, degrees of freedom = 5, p = 0.6322). Converting the Chi-square statistics to Cohen’s effect sizes yielded w = 0.0315 (95% confidence interval (0, 0.0645)) for sex at birth and outcome options and w = 0.0453 (95% confidence interval (0, 0.0716)) for sex at birth and gap explanations, indicating very small effect sizes.

By contrast, Pearson’s chi-square tests demonstrated statistically significant associations between age group and outcome options (χ² = 30.3958, degrees of freedom = 9, p = 0.0004) and between age group and gap explanations (χ² = 56.3943, degrees of freedom = 15, p < 0.0001). Cohen’s effect sizes were w = 0.1274 (95% confidence interval (0.0579, 0.1576)) for age group and outcome options and w = 0.1832 (95% confidence interval (0.1059, 0.2097)) for age group and gap explanations, representing small and small-to-moderate effects, respectively.

## Discussion

This study evaluated adherence to colorectal cancer screening guidelines in an FQHC population. Most patients identified as lacking appropriate colorectal cancer screening were older adults aged 65-75 years, particularly women, whereas the lowest proportion occurred among newly eligible patients aged 45-55 years. Although more than half of the total population could not be reached, more than half of the 585 patients who were successfully contacted agreed to complete screening, demonstrating that proactive outreach can meaningfully improve adherence. Documentation discrepancies within the electronic medical record accounted for an additional 243 cases initially classified as unscreened, underscoring the need for improved documentation and data accuracy in population health reporting.

Our findings are consistent with prior reports highlighting disparities in colorectal cancer screening among underserved and minority populations [11]. Similar to prior studies, women in older age groups were less likely to maintain up-to-date screening [13]. This may reflect both patient perceptions that prior screening was sufficient and decreased vigilance by providers in recommending repeat colonoscopy after 10 years. Newly eligible patients aged 45-55 years were least likely to be flagged as unscreened, a finding likely related to provider awareness of the U.S. Preventive Services Task Force 2021 guideline update lowering the screening age from 50 to 45 years [5]. A similar trend was reported in national data, where adherence to colorectal cancer screening increased after the recommendation change, particularly among younger adults [9].

Our findings further underscore significant limitations in electronic medical record documentation and population health reporting. Although patients who were ultimately confirmed to have up-to-date screening were excluded from the statistical analysis, many of these individuals had been misclassified as overdue based on electronic medical records and population health data. In several cases, screening had been completed outside the system or was not accurately documented, requiring direct patient confirmation to identify true screening status. This misclassification highlights a broader and well-recognized problem in which incomplete data capture undermines the accuracy of EMR-based quality measures and population health metrics. This problem has been noted in other studies, where gaps in data mapping reduced the reliability of screening metrics [14]. 

Barriers to colorectal cancer screening in underserved populations include lack of insurance, limited patient education, and competing health priorities during clinic visits [18]. The findings from this study suggest that direct outreach outside of routine visits may help address these barriers. Importantly, more than half of the contacted patients, 319 (55%), accepted screening, even when they had previously refused in clinic settings. Another barrier involves the type of screening offered. Prior research has demonstrated lower adherence to fecal immunochemical test screening compared with colonoscopy because of its annual requirement [20]. In this population, uninsured patients were primarily offered fecal immunochemical testing, which may partially explain the refusal rate of 266 (45%) among those contacted. 

The results highlight several important implications. Proactive outreach can substantially improve screening adherence. Older women may represent a priority group for targeted education and reminders. Accurate electronic medical record documentation is essential for valid quality reporting. Finally, insurance coverage and screening modality strongly influence adherence, with uninsured patients facing additional challenges.

This study has several limitations. More than half of the patients could not be reached, which limits the generalizability of the findings. It was not possible to verify whether some patients completed screening after being contacted, but without updating their electronic medical record. This was a single-center study, and results may not apply to other populations. Finally, statistical analyses were limited to descriptive and comparative methods; future research using multivariate approaches may better identify predictors of adherence.

## Conclusions

This project engaged the patient population at CHC to thoroughly evaluate adherence to colon cancer screening guidelines. Our primary objective was to identify patients who fit the established screening criteria, specifically individuals between the ages of 45 and 75 who had not received appropriate colon cancer screening within the previous year. One noteworthy discovery from our study was that the most frequently identified patients lacking adequate colon cancer screening were females aged 65 to 75 years. In contrast, we observed that the lowest percentage of individuals not screened properly was those newly eligible, the age group 45 to 55. This suggests that our team has effectively pinpointed patients for screening as they first meet the recommended age requirement, thereby improving the early identification of those at risk. Among the patients we were able to contact successfully, we found that a substantial majority opted to undergo colon cancer screening. This outcome indicates that, with increased effort and a greater allocation of time, clinics can improve their colon cancer screening efforts. These findings also highlight the critical importance of maintaining an ongoing dialogue about colon cancer screening with patients. While our study offers valuable insights into screening adherence and the reasons for non-adherence, the limitations we encountered, such as issues related to insurance coverage, the patient follow-through of screening orders placed, and proper EMR documentation of screening outcomes, underscore the need for further research.
